# Engineered M2a macrophages for the treatment of osteoarthritis

**DOI:** 10.3389/fimmu.2022.1054938

**Published:** 2022-12-13

**Authors:** Chi Liang, Song Wu, Guang Xia, Junjie Huang, Zi Wen, Wenxiu Zhang, Xu Cao

**Affiliations:** ^1^ Department of Orthopaedics of the 3rd Xiangya Hospital, Central South University, Changsha, China; ^2^ Institute of Basic Medicine and Cancer (IBMC), Chinese Academy of Sciences, Changsha, China

**Keywords:** macrophages, Cas9-ribonuclear proteins, cell therapy, tumor necrosis factor receptor 1, osteoarthritis

## Abstract

**Background:**

Macrophage is a central regulator of innate immunity. Its M2 subsets, such as interstitial synovial macrophages, have been found to play critical roles in suppressing chronic inflammation and maintaining homeostasis within the joint. These macrophages have great potential as a disease-modifying cell therapy for osteoarthritis (OA). However, this has not yet been studied.

**Methods:**

Macrophages were isolated from the bone marrow of rats. We constructed a stable macrophage that “locked” in anti-inflammatory and pro-regenerative M2a polarity (L-M2a) by simultaneously knocking out tumor necrosis factor receptor 1 (TNFR1) and overexpressing IL-4 using Cas9-ribonuclear proteins (Cas9-RNP) and electroporation. *In vitro*, these L-M2a macrophages were treated with OA synovial fluid or co-cultured with OA chondrocytes or fibroblast-like synoviocytes (FLS). *In vivo*, L-M2a macrophages were injected intra-articularly to evaluate their homing and engrafting abilities and therapeutic effects on OA progression using a rat model.

**Results:**

L-M2a macrophages displayed a typical anti-inflammatory phenotype similar to that of M2 macrophages *in vitro*. In OA microenvironment, L-M2a macrophages maintained a stable anti-inflammatory phenotype, whereas unmodified M2 macrophages lost their phenotype and switched to M1 polarity. L-M2a macrophages demonstrated a potent anti-inflammatory effect in crosstalk with OA-FLSs and an anti-degenerative effect in crosstalk with senescent OA chondrocytes. *In vivo*, compared with M2 macrophages and exosomes, L-M2a macrophages exhibited significantly superior therapeutic effects in OA by successfully resolving inflammation, restoring tissue homeostasis, and promoting cartilage regeneration.

**Conclusion:**

The engineered L-M2a macrophages maintained a superior anti-inflammatory and pro-regenerative capacity in the inflammatory OA microenvironment and represents an ideal new strategy for the disease-modifying therapy of OA.

## Introduction

Osteoarthritis (OA) is viewed as a multifactorial disorder affecting the entire joint, in which persistent low-grade inflammation plays a central role. Due to the interaction between immune system and various factors (including damage associated molecules and metabolic dysfunction), this long-term inflammation is initiated in the early stage of OA, leading to cartilage loss and progressive joint degeneration (1). Although disease-modifying OA drugs targeting this pathomechanism have been developed in recent years, no drug has yet achieved satisfactory results capable of delaying the clinical progression of OA (2).

Macrophage (Mφ) is a primary immunomodulator of the innate immune system and critical regulator of chronic inflammation and tissue homeostasis. Based on the activation pattern, Mφs have been typically dichotomized into two phenotypes: M1-polarized Mφs (‘classically’ activated) are pro-inflammatory with the release of interleukin (IL)-1β, IL-6, IL-12, IL-23, and TNF-α, while M2-polarized Mφs (‘alternatively’ activated) are anti-inflammatory and pro-regenerative, secreting IL-4, IL-10, and TGF-β, etc (3). Specific Mφ phenotypes differentially modulate the inflammation and regeneration responses of multiple cell types during the onset or progression of OA (4). M1 Mφs stimulate the production of inflammatory cytokines and matrix-degrading enzymes, promoting hypertrophic differentiation of chondrocytes and cartilage destruction (5). In contrast, M2 Mφs are characterized by pro-chondrogenic genes expression, which improves cartilage health (6). Recently, studies have observed a subpopulation of RELMα^+^ interstitial synovial macrophages (ISMφ) with a stable M2a phenotype in the sub-lining layer of synovium (7). These M2a ISMφs release resolvins to switch fibroblasts from a proinflammatory to a reparative state to restore homeostasis, and were proposed to act as critical cellular checkpoints and off switches for intra-articular inflammation (8). In OA, an increase in M1 and a corresponding decrease in M2 Mφ have been observed to be directly related to OA severity (5, 9).

M2 Mφ possesses potent anti-inflammatory and pro-regenerative properties, and intrinsic ‘homing’ ability to migrate to sites of injury or inflammation (10). Therefore, it has great potential as an ideal seed cell for disease-modifying cell therapy for OA. However, to our knowledge, transplantation of M2 Mφ has not been studied in the treatment of OA. A major obstacle hindering the application of exogenous Mφ in regenerative medicine lies in the plasticity of its polarity. The polarization of Mφs is not fixed and characterized by high adaptability and plasticity in the microenvironment (11). Studies have found that the new environment is sufficient to reprogram well-polarized Mφs after transplantation (12, 13). This may lead to the loss of phenotype and expected function of the delivered Mφs. For example, Qi et al. observed the polarization loss of M2 Mφs in the inflammatory environment after transplantation, which impaired their protective effect against renal inflammatory injury (12). Thus, constructing M2 Mφs with a stable or ‘locked’ phenotype will provide a great opportunity for the treatment of inflammatory diseases including OA.

As tumor necrosis factor (TNF) is a major anti-M2 (pro-M1) factor in the chronic inflammatory microenvironment, such as OA, and IL-4 is a major pro-M2a factor (3), we proposed a stable Mφ that, like ISMφ, is ‘locked’ in M2a phenotype by knocking out TNFR1 and overexpressing IL-4 using Cas9-ribonuclear protein (Cas9-RNP) complexes and electroporation. In this study, we attempted to treat OA by intra-articular delivery of exogenous M2a Mφs for the first time and investigated the effect of this ‘locked M2a-like Mφ’ (L-M2a Mφ) on restoring the homeostasis of OA cartilage and synovium. We concluded that this L-M2a Mφ successfully maintained a stable phenotype in OA environment and significantly alleviated inflammation, promoted regeneration and delayed progression of OA in animal models.

## Materials and methods

All experiments involving human tissues and animals were performed in accordance with guidelines approved by the Institutional Review Board (IRB). The source and identifier of reagents in this study are showed in **Supplementary Table S1**.

### Isolation and polarization of bone marrow derived Mφ

All the Sprague-Dawley (SD) rats used in this study were provided by the Department of Laboratory Animals of the Central South University (Changsha, China). The bone marrow was harvested from the tibia and femurs of the rats. Red blood cells were lysed with ammonium-chloride-potassium lysis buffer. The remaining bone marrow cells were plated in RPMI-1640 medium with 10% FCS and 100 U/100 µg/ml penicillin/streptomycin supplemented with 50 ng/ml M-CSF at a density of 1 × 10^6^ cells/ml for 5 days to obtain M0 Mφs. Subsequently, the Mφ cells were treated with IFNγ (20 ng/ml) and LPS (100 ng/ml), or IL-4/IL-13 (each 10 ng/ml) for 24 h to obtain M1 and M2 Mφ, and the cytokine secretion of M0, M1, and M2 were examined.

### Construction of LM2a Mφ

EasyEdit sgTNFR1 and NLS-Cas9-EGFP nuclease proteins were purchased from GenScript (Nanjing, China). The pCDNA-IL-4 plasmid was purchased from Tsingke Biotechnology Co. Ltd (Shanghai, China). LM2a Mφ with nucleofection was constructed according to the manufacturers and a previous study’s instructions (14). Briefly, day 4 BMDMs were resuspended in 100 µL nucleofection solutions at a density of 1 × 10^6^ cells per reaction in and mixed with 1 Cas9-RNP (25 µM Cas9 protein premixed with 100 µM sgRNA at a ratio of 1:3 for 10 min) and 2 µg IL-4 plasmid. The suspension was transferred into a certified cuvette and inserted into Nucleofector^®^ Cuvette Holder using the Nucleofector^®^ Program Y-001.

Nucleofected Mφs were cultured in prewarmed RPMI medium supplemented with 10% FBS for 48 h and treated with IL-4/IL-13 (each 10 ng/ml) for 24 h. LM2a Mφs were assessed using flow cytometry, DNA cleavage assay, and western blotting.

### DNA cleavage assay

gDNA was extracted from LM2a and M2 Mφs using QuickExtract solution, following the manufacturer’s instructions. DNA primers were generated upstream and downstream of the sgRNA target editing site (sequences are presented in **Supplementary Table S2**). PCR was run for 30 cycles following the manufacturer’s instructions, and the products were determined with 1% agarose gel electrophoresis and analyzed using Gelation imaging system (ImageQuant350, GE, USA).

### Flow cytometry analysis

Polarized and constructed Mφs were suspended in flow cytometry staining buffer (phosphate-buffered saline with 1% fetal calf serum). Cells were treated with CD86 (1:100) rabbit, CD206 (1:100) mouse, and IgG isotype control (1:100) primary antibody, and then stained with Alexa Fluor 488 anti-Rabbit IgG antibody (1: 500) and Alexa Fluor 405 anti-Mouse IgG antibody (1: 500) respectively. All samples were detected by Becton Dickinson FACScan (BD Biosciences) and analyzed with FlowJo10. The gating strategy was showed in **Supplementary Figure S1B**.

### *In vitro* stimulation assay

Human OA synovial fluid (OASF) was aspirated from the knee joints of five patients with OA at the Third Xiangya Hospital of the Central South University and used to mimic the joint microenvironment of OA. Detailed clinical information and Kellgren–Lawrence gradation of all participants was presented in **Supplementary Table S3**. OASF was further purified by centrifugation at 3000 rpm for 15 min, and the supernatants were harvested. M2, and L-M2a Mφs were cultured in RPMI1640 or diluted OASF (50%) for 3 days. Polarized and inflammatory phenotypes were detected by RNA-sequencing, western blotting, RT-qPCR, immunofluorescence, and flow cytometry.

### Co-culture assay

A co-culture system was established using six-well transwell plates. OA chondrocytes and FLS were isolated and cultured using a previously described method from a rat OA model (15), which was established by Hulth’s surgery. FLS (1 × 10^6^) or chondrocytes (1 × 10^6^) were cultured in the lower compartments, and 1 × 10^6^ M2a or LM2a Mφs were cultured in the upper compartments in RPMI 1640 with 10% FBS. FLS, chondrocytes, M2a, or LM2a Mφs were cultured alone as controls. Co-cultures were maintained for 1 or 7 days before evaluation. The anti-inflammatory factors and polarization markers of M2a and LM2a were analyzed using western blotting and RT-qPCR. The inflammatory factors and invasion ability of FLS were evaluated using western blotting, RT-qPCR, and invasion assays. The degenerative indicators of chondrocytes were determined using western blotting and RT-qPCR. The number of senescent chondrocytes was evaluated by RT-qPCR using p16^INK4a^ and p21^Cip1^ and SA-β-gal staining kits. Chondrocyte apoptosis rates were analyzed by annexin V-FITC/propidium iodide (PI) double staining according to the manufacturer’s instructions. Co-cultures were conducted in technical triplicate for each assay, and five random fields of each well were selected for evaluation.

### Mφ tracing *in vivo*

Hulth’s surgery was performed on the right joints of 12-week-old male rats to establish an OA model (16). Overall, 20 μl 1× 10^6^/ml M2 or LM2a (n= 6 for each group) were labelled with CellTracker CM-Dil according to the manufacturer’s protocol and then injected intra-articularly at 4 weeks after surgery. Mφ viability was examined using an *in vivo* imaging system (IVIS Lumina II, Caliper Life Science, USA) at 1, 2, and 4 weeks after injection. At 1 and 4 weeks after the injection, the rats were sacrificed. The samples were decalcified in JYBL I for 48 h, cut into frozen sections, and stained by immunofluorescence for CD206, which was detected by fluorescence microscopy (Leica TCS-SP5, DM6000-CFS).

### Histological evaluation

For the histological evaluation, 20μl PBS (OA group), 1× 10^6^/ml M2a, LM2a, or M2a exosomes derived from equal Mφs (n= 5 for each group) were injected intra-articularly 4 weeks after the Hulth’s surgery. The sham operation group was used as control. After 8 weeks, the rats were sacrificed. Joint samples were collected and fixed in 4% paraformaldehyde for 48 h. The samples derived from the rats were detected using magnetic resonance imaging (7.0T MRI Biospoin GmbH, BRUKER) and graded using the MOAKS score. Synovial samples were collected from each group for western blot analysis. All samples were decalcified in 0.5M EDTA for 4 weeks, embedded in paraffin, and cut into sections (6 μm). Immunohistochemistry and immunofluorescence were performed using anti-MMP13, anti-IL-6, and anti-Col I antibodies. Secondary antibodies were detected using a fluorescent secondary antibody or rabbit streptavidin-biotin detection system kit according to the manufacturer’s protocol. Slices of rat knee joints were stained with safranin O/fast green. The Osteoarthritis Research Society International (OARSI) scoring system was used to evaluate OA cartilage pathology.

### Western blot analysis

Total proteins obtained from the cells and tissues were subjected to SDS–PAGE, transferred to PVDF membranes, and blocked in 5% skimmed milk for 30 min. The membranes were incubated overnight at 4°C with primary antibodies against CD206 (1:1000), CD86 (1:1000), iNOS (1:1000), TNFR1 (1:1000), IL-4 (1:1000), MMP13 (1:1000), IL-6 (1:1000), Col I (1:1000), Col II (1:1000), Col X (1:1000), ACAN (1:1000), p-STAT6 (1:500), STAT6 (1:1000), N-cadherin (1:1000), and GAPDH (1:8000). The membranes were incubated with HRP-conjugated secondary antibodies (1:10000) at temperature for 1 h and developed using electrochemiluminescence western blot reagents. Then the membranes were analyzed using a UVP Chem studio PLUS 815 (Analytik Jena, Germany).

### RT-qPCR

Total RNA was isolated from the synovium or cells using TRIzol reagent and the consentration was measured by a NanoPhotometer spectrophotometer (IMPLEN, CA). RNA was then converted to cDNA according to the manufacturer’s instructions. ChamQ Universal SYBR qPCR Master Mix was used for qPCR. Gene transcription levels were normalized to those of GAPDH. The primer design is shown in **Supplementary Table S2**.

### Immunofluorescence assay

Mφ or slices were fixed in 4% paraformaldehyde. BSA (4%) was used to block non-specific binding. The cells were then incubated with CD86, CD206, and CD4 primary antibodies overnight. Fluorescent secondary antibodies were used, and the samples were subsequently stained with 4,6-diamidino-2-phenylindole (DAPI) for 5 min. The cells or slices were observed under a confocal fluorescence microscope.

### RNA sequencing

Total RNA was extracted using Trizol reagent (thermofisher, 15596018) following the manufacturer’s procedure. After total RNA was extracted, mRNA was purified from total RNA (5ug) using Dynabeads Oligo (dT) (Thermo Fisher, CA, USA) with two rounds of purification. Following purification, the mRNA was fragmented into short fragments using divalent cations under elevated temperature (Magnesium RNA Fragmentation Module (NEB, cat.e6150, USA) under 94°C 5-7min). Then the cleaved RNA fragments were reverse-transcribed to create the cDNA by SuperScript™ II Reverse Transcriptase (Invitrogen, cat.1896649, USA), which were next used to synthesise U-labeled second-stranded DNAs with E. coli DNA polymerase I (NEB, cat.m0209, USA), RNase H (NEB, cat.m0297, USA) and dUTP Solution (Thermo Fisher, cat.R0133, USA). An A-base was then added to the blunt ends of each strand, preparing them for ligation to the indexed adapters. Each adapter contained a T-base overhang for ligating the adapter to the A-tailed fragmented DNA. Dual-index adapters were ligated to the fragments, and size selection was performed with AMPureXP beads. After the heat-labile UDG enzyme (NEB, cat.m0280, USA) treatment of the U-labeled second-stranded DNAs, the ligated products were amplified with PCR. At last, we performed the 2×150bp paired-end sequencing (PE150) on an Illumina Novaseq™ 6000 following the vendor’s recommended protocol.

Genes differential expression analysis was performed by DESeq2 software between two different groups (and by edgeR between two samples). The genes with the parameter of false discovery rate (FDR) below 0.05 and absolute fold change ≥ 2 were considered differentially expressed genes. Differentially expressed genes were then subjected to enrichment analysis of GO functions and KEGG pathways. Principal component analysis (PCA) and correlation analysis were performed with princomp function of R (http://www.r-project.org/).

### Statistical analysis

All experiments were repeated at least three times, and the data are presented mean with 95% confidence intervals (CI) by individual dot plots. A one-way analysis of variance (ANOVA) was used for comparisons across multiple groups including phenotypic identification and *in vivo* experiments, while Dunnett’s test was used for *post-hoc* multiple comparisons. Comparisons of M2 and L-M2a Mφs *in vitro* were calculated using a two-way ANOVA followed by Tukey’s multiple comparisons test. All data analyses were performed using the GraphPad Prism 8.

## Results

### Construction and phenotypic evaluation of genome edited L-M2a Mφ

**Figure 1:**
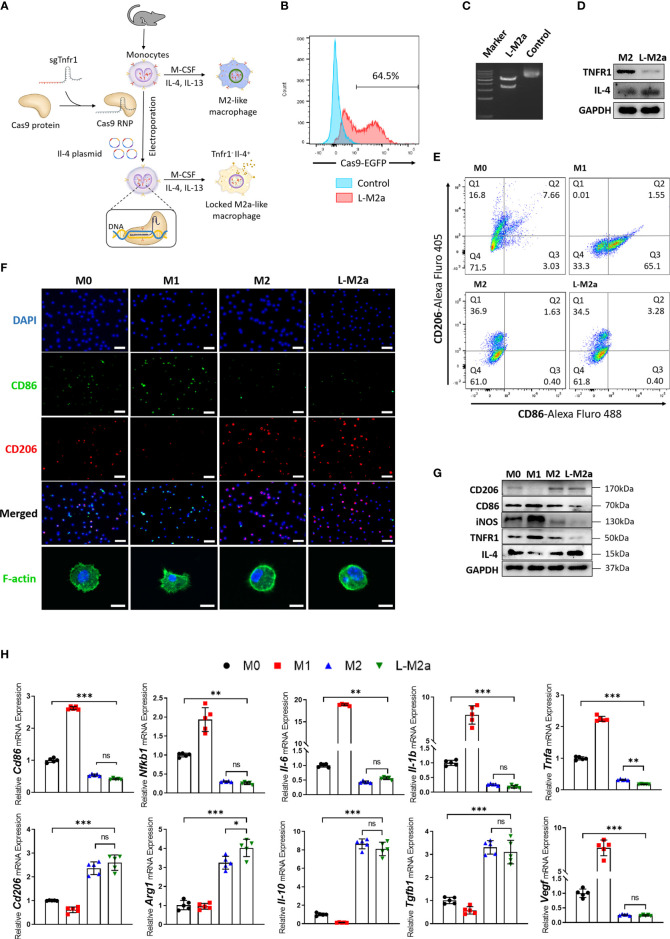
Construction and identification of L-M2a Mφ. **(A)** Illustration of the polarization of M0, M1, M2 Mφ and construction of L-M2a Mφ. **(B-D)** Validation of knockout efficiency of sgTNFR1-EGFP-Cas9-RNP and overexpression of pCDNA -IL-4 using flow cytometry **(B)**, DNA cleavage assay **(C)** and western blot **(D)**. **(E-H)** Polarized phenotype, inflammatory phenotype and morphological differences among M0, M1, M2a, L-M2a Mφ by western blot (E, n=3), immunofluorescence (F, upper part, bar= 100 μm), confocal (F, bottom part, bar=10 μm), flow cytometry **(G)** and RT-qPCR (H, n=5). ∗p < 0.05; ∗∗p < 0.01; ∗∗∗p < 0.001. ns, no significance; Mφ, macrophage; L-M2a Mφ, locked M2a macrophage.

Our CRISPR-Cas9 genome editing strategy for Mφ is outlined in **Figure 1A**. The knockout efficiency of TNFR1 was shown to be more than 60% by flow cytometry (**Figure 1B**), DNA cleavage assay (**Figure 1C**), and western blot (**Figure 1D**), while the overexpression efficiency of IL-4 is shown in **Figure 1D**. The feasibility of this editing strategy was also verified with human-derived primary Mφs (**Supplementary Figure S1A**). We examined the phenotype of Mφs *in vitro* using immunofluorescence and flow cytometry. In contrast to M0 and M1 Mφ, which variably express the M1 marker CD86, both M2 and genome-edited L-M2a Mφs were characterized by low expression of CD86 and high expression of the M2 marker CD206 *in vitro* (**Figures 1E, F**). From the aspect of morphology, M1 Mφ showed the characteristic changes of flattening and a lot of pseudopodia (**Figure 1F**). Western blotting suggested lower CD86 levels in L-M2a Mφs than in M2 Mφs (**Figure 1G**). L-M2a Mφs displayed a typical anti-inflammatory phenotype similar to that of M2 Mφs *in vitro*, with high expression of IL-4, IL-10, TGF-β, and Arginase-1, and low expression of IL-1β, IL-6, TNFα, iNOS, NF-κB, and VEGF (**Figures 1G, H**). In addition, the same gene editing strategy in M1 Mφs can not be sufficient to reverse the M1 phenotype (**Figure S3B**).

### L-M2a Mφ maintains a stable polarized phenotype in the OA microenvironment

**Figure 2:**
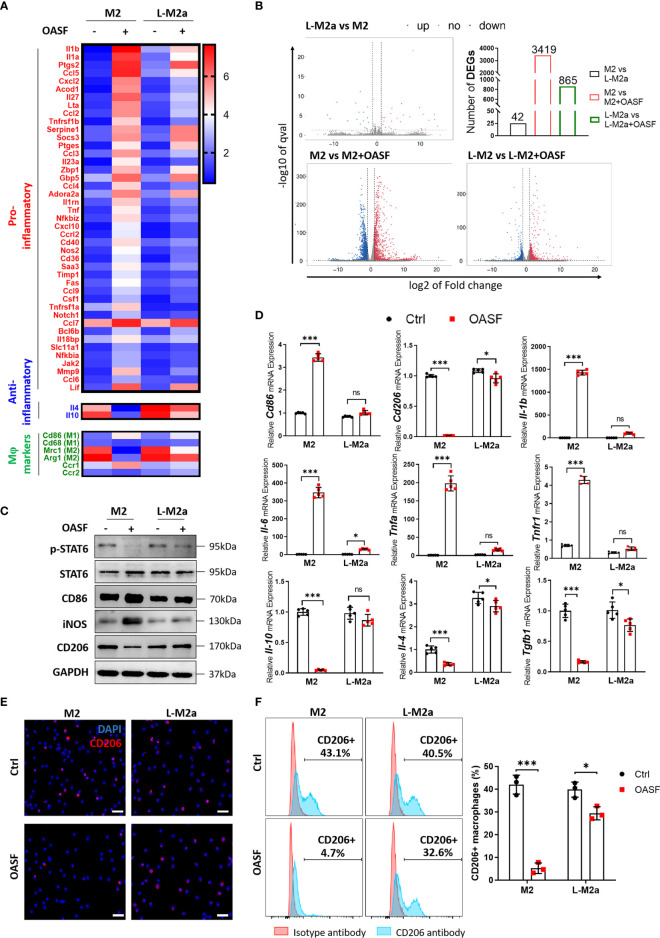
L-M2a Mφs maintained a more stable polarization in the presence of OASF *in vitro*. **(A, B)** A heatmap about inflammatory related transcriptome changes **(A)** and number of differentially expressed genes **(B)** among M2 and L-M2a macrophages treated with or without OASF by RNA sequencing (n=3). **(C, D)** Validation of expression level of saveral typical genes in **(A)** using western blot (C, n=3) and RT-qPCR (D, n=5). **(E, F)** Expression of M2 maker CD206 in M2, and L-M2a Mφ when stimulated with or without OASF by immunofluorescence (E, bar= 100μm) and flow cytometry **(F)**. ∗p < 0.05; ∗∗∗p < 0.001. ns, no significance; Mφ, macrophage; L-M2a Mφ, locked M2a macrophage; OASF, OA synovial fluid.

To investigate whether L-M2a Mφs had a more stable phenotype in the OA microenvironment, we analyzed the transcriptome changes of M2 and L-M2a Mφs treated with OA synovial fluid (OASF) by RNA-sequencing (**2A, B**, **S1C**). We found that under the treatment of OASF, M2 Mφs exhibited up-regulation of a variety of pro-inflammatory cytokines and chemokines (Il1b, Il6, Tnfa, Ccl5, Ccl2, Ccl9, Cxcl2), while down-regulation of M2 polarization markers (Mrc1, Arg1) and anti-inflammatory cytokines (Il10, Il4). In contrast, L-M2a Mφs showed obvious resistance to OASF and remained a more stable expression of M2 polarization markers and anti-inflammatory cytokines (**Figure 2A**). Meanwhile, we used the number of differentially expressed genes (DEGs) in macrophages to reflect the extent to which they were affected by OASF. Compared with the 3419 DEGs of M2 Mφs, L-M2a Mφs only had 865 DEGs after OASF treatment, which indicated a more stable phenotype of L-M2a Mφs in OA microenvironment (**Figure 2B**). Western blot and RT-qPCR verified that the expression of CD86 and CD206 in L-M2a Mφs was almost unaffected, whereas it was significantly changed in M2 Mφs (**Figures 2C, D**, **S3F**). Similarly, the ratio of CD206hi cells remained stable in L-M2a Mφs (from 40.5%−32.6%), whereas it decreased dramatically in M2 (from 43.1%−4.7%) Mφs (**Figures 2E, F**). OA-SF induced the expression of pro-inflammatory factors (IL-1β, IL-6, TNF-α and iNOS) and inhibited the activation of anti-inflammatory IL-4/STAT6 signaling in M2 Mφs. In contrast, L-M2a Mφs were less affected and maintained an anti-inflammatory phenotype (**Figure 2**). However, L-M2a Mφs didn’t show the same stability against the stimulation of LPS+IFNγ (**Figure S3C**).

### L-M2a Mφs have potent anti-inflammatory effects in crosstalk with OA-FLS

**Figure 3:**
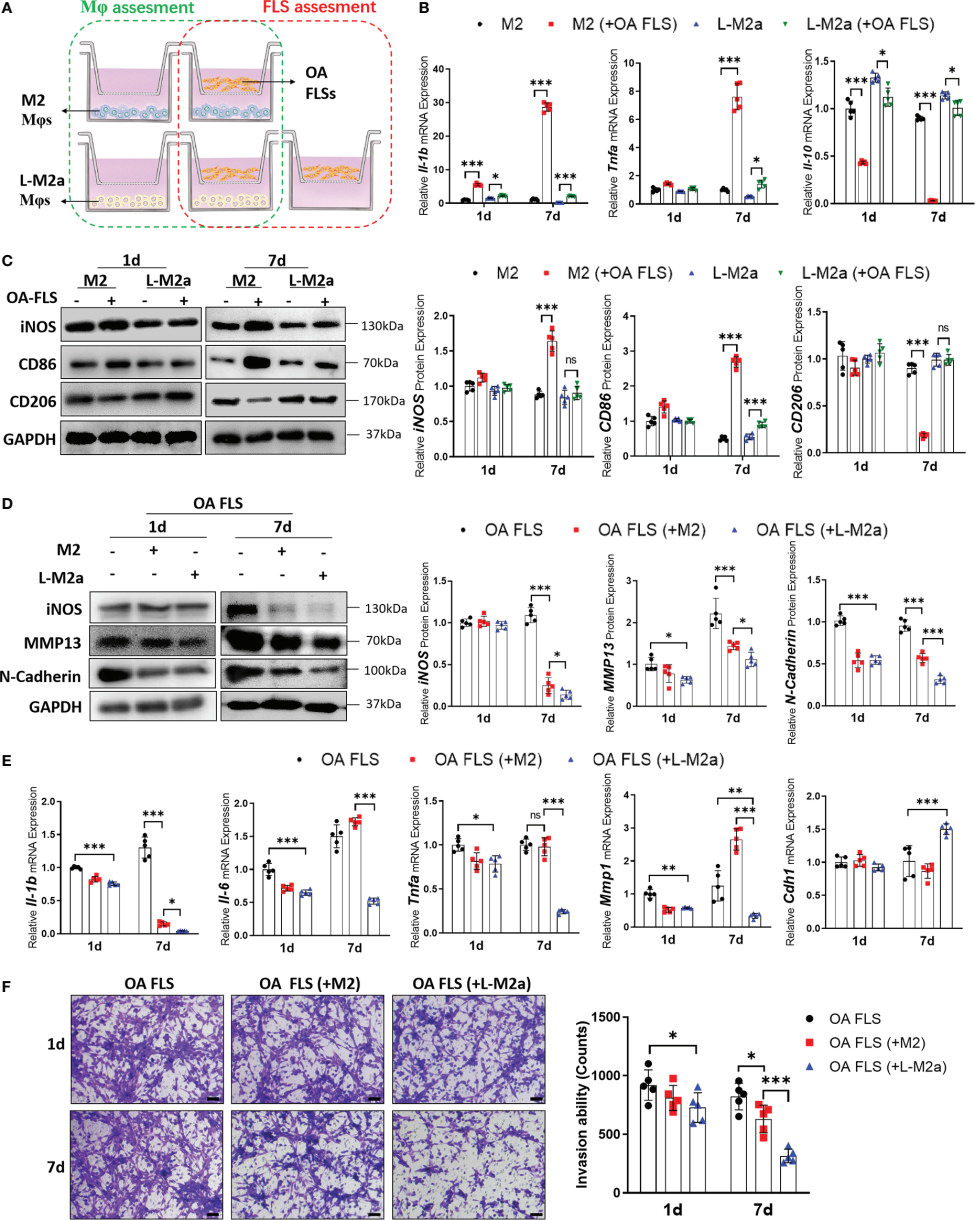
L-M2a Mφ had potent anti-inflammatory effects in crosstalk with OA-FLS. **(A)** Illustration of transwell co-culture system of Mφs and OA FLSs. **(B, C)** Polarized phenotype and inflammatory phenotype of M2 and L-M2a Mφ co-cultured with or without OA FLS for 1 or 7 days using RT-qPCR (B, n=5) and Western blot (C, n=3). **(D, F)** Assessment of EMT markers **(D)**, inflammation factors **(D, E)** and invasion activity (F, bar= 100μm) in OA-FLS co-cultured with control, M2 or L-M2a Mφ. ∗p < 0.05; ∗∗p < 0.01; ∗∗∗p < 0.001. ns, no significance; Mφ, macrophage; L-M2a Mφ, locked M2a macrophage; FLS, fibroblast-like synoviocyte.

We established a transwell co-culture system *in vitro* for M2/L-M2a Mφs and OA-FLS (**Figure 3A**). After co-culture with OA-FLS, M2 Mφs showed a trend of transition to the M1 phenotype, with decreased polarization markers of M2 (CD206, ARG1). This trend was more pronounced with prolonged coculture. On day 7, M2 Mφ shifted to an obvious pro-inflammatory state (TNFα and IL-β expression) and M1-polarized phenotype (CD86 and CD206 expression). In contrast, L-M2a Mφs showed a more stable anti-inflammatory state and polarization marker expression in co-culture with OA-FLS (**Figures 3B, C**). Moreover, M2 Mφs exhibited a limited ameliorative effect on the pro-inflammatory and destructive phenotype of OA-FLS, with only mild inhibition of IL-1β and N-cadherin expression and FLS invasion at day 7. In contrast, L-M2a Mφ significantly inhibited the pro-inflammatory factors (IL-1β, IL-6 and TNFα) expression and invasive activity (MMP1 and transwell invasion) of OA-FLS (**Figures 3D–F**).

### L-M2a Mφ exhibits anti-degenerative effects through crosstalk with OA chondrocytes

**Figure 4:**
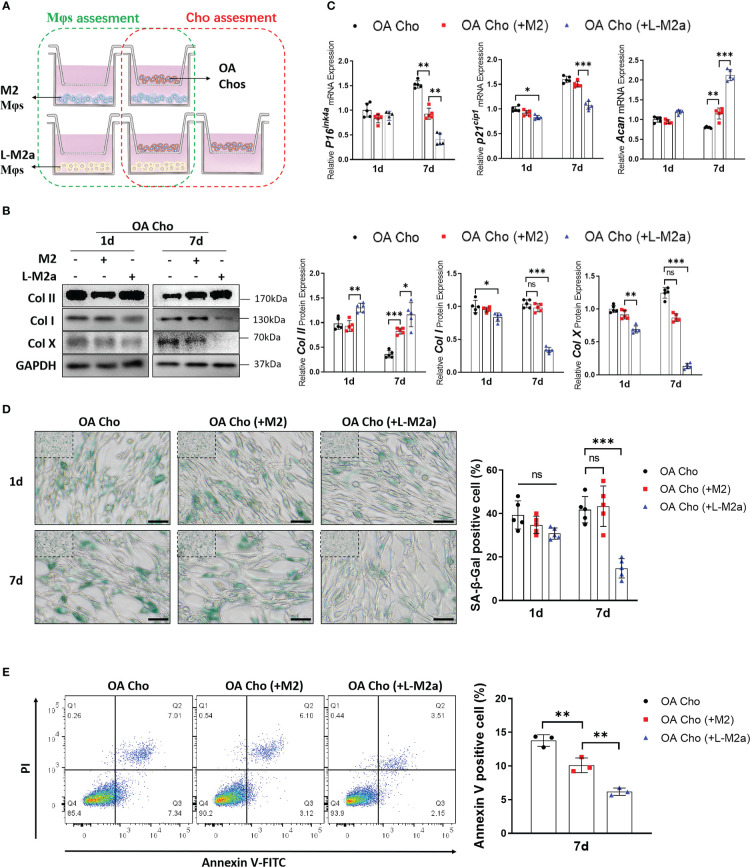
L-M2a Mφs exhibited anti-degenerative effects in crosstalk with OA-chondrocytes. **(A)** Illustration of transwell co-culture system of Mφs and OA chondrocytes. **(B, C)** Assessment of collagen synthesis ability **(B)**, inflammation factors and senescence markers **(C)** in OA chondrocytes co-cultured with control, M2 or L-M2 cells for 1 and 7 days. **(D)** Staining of SA-β-Gal (green, bar= 100μm) in OA chondrocytes co-cultured with control, M2 or L-M2a Mφs for 1 and 7 days. **(E)** Apoptosis rates for different Mφ-stimulated OA chondrocytes after 7 days coculture were analyzed by flow cytometry using annexin V-FITC/PI apoptosis analysis. ∗p < 0.05; ∗∗p < 0.01; ∗∗∗p < 0.001. ns, no significance; Mφ, macrophage; L-M2a Mφ, locked M2a macrophage; Cho, chondrocyte.

We established an *in vitro* transwell co-culture system for M2/L-M2a Mφs and OA-derived chondrocytes (OA-Cho) (**Figure 4A**). The changing trend in the phenotype of Mφs was similar to that observed in the OA-FLS co-culture described above (**Supplementary Figures S2A, B**). For OA-Chos, M2 Mφs exhibited only a slight ameliorative effect on several degenerative indicators (Col I, Col X, and ACAN). In contrast, L-M2a Mφs markedly improved the degenerative phenotype of OA-Chos (increased hyaline cartilage marker Col II and ACAN, and decreased fibrocartilage marker Col I and hypertrophic marker Col X) and alleviated cellular senescence (decreased p16INK4a and p21Cip1 expression and SA-β-Gal staining) (**Figures 4B,– D**) and apoptosis (decreased Annexin V positive cells) (**Figure 4E**) in the co-culture system.

### L-M2a Mφ restored tissue homeostasis and promoted cartilage regeneration in a rat OA model

**Figure 5:**
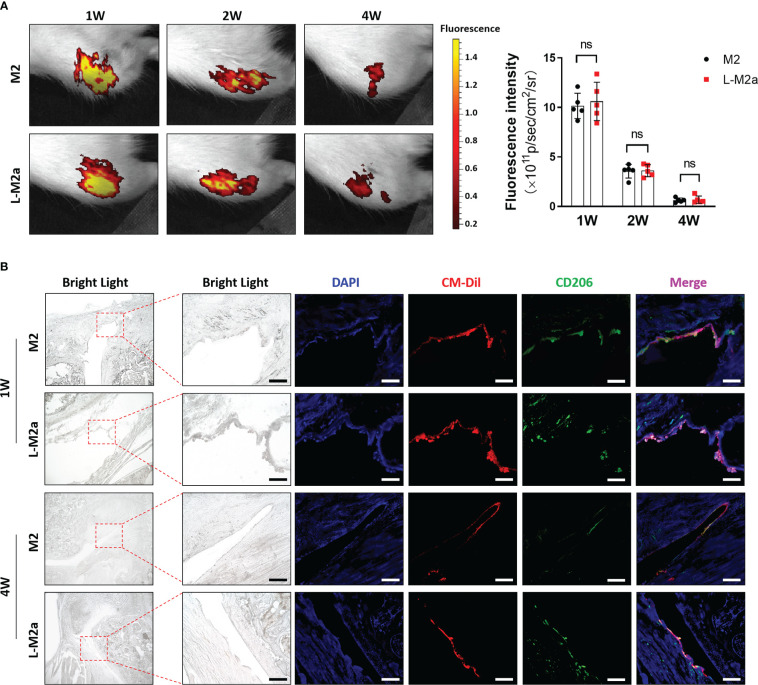
Tracing of L-M2a Mφ *in vivo*. **(A)** The fluorescence intensity of CM-Dil labelled M2 or L-M2a Mφ at 1, 2, and 4 weeks after intra-articular injection by IVIS. **(B)** The bright light, DAPI staining (blue), CM-Dil labelled M2 or L-M2a Mφ (red), CD206 immunofluorescence (green) and merge vision of joints at 1 and 4 weeks after intra-articular injection. bar= 100μm. ns, no significance; Mφ, macrophage; L-M2a Mφ, locked M2a macrophage.

We used the Hulth’s method to establish an OA model in SD rats. M2 or L-M2a Mφ (labelled with CM-Dil) was injected intra-articularly 4 weeks after surgery. *In vivo* imaging systems (IVIS) showed strong fluorescence intensity in the knee joints 4 weeks after injection, whereas there was no statistical difference between M2 and L-M2a Mφs (**Figure 5A**). At week 1 after injection, Mφs in both groups homed to the OA synovium and maintained the M2 phenotype (CD206). However, M2 Mφs rapidly lost their M2 phenotype within 4 weeks, while L-M2a Mφs maintained a high positivity for CD206 (**Figure 5B**).

**Figure 6:**
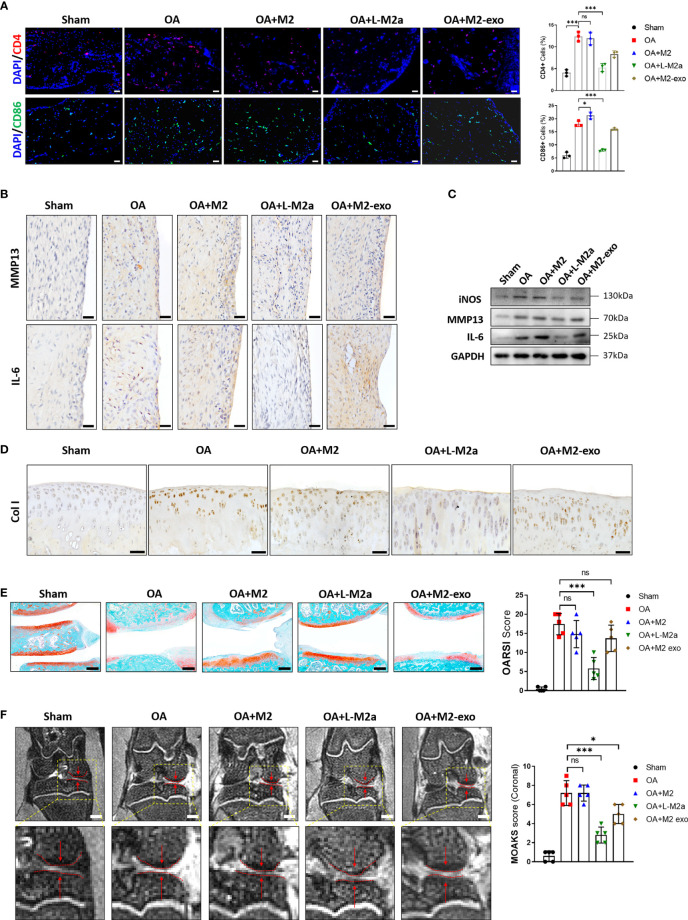
L-M2a Mφs promoted OA repair in a rat OA model. **(A)** Immunofluorescence of CD4+ (red) and CD86+ (green) cells and DAPI staining (blue) in Sham, OA, M2, L-M2a, and M2-exo groups at 4 weeks after injection (n=3, bar= 100μm). **(B, C)** Immunohistochemical staining (B, bar= 100μm) and western blot **(C)** of IL-6, MMP13, and iNOS in synovium of rat joints. **(D)** Immunohistochemical staining of Col I in cartilage of rat joints. bar= 100μm. **(E)** Safranin O/fast green staining of cartilage (left) and OARSI score (right) (n=5, bar=500μm). **(F)** Medial joint space (left, red arrow between two dotted lines) in coronal position of total knee and MOAKS score (right) by magnetic resonance imaging. (n=5, bar=1000μm). ∗p < 0.05; ∗∗∗p < 0.001. ns, no significance; Mφ, macrophage; L-M2a Mφ, locked M2a macrophage; M2-exo, M2 macrophage exosomes.

Histological evaluation of OA was performed 8 weeks after surgery. OA modeling induced immune cell (CD4^+^ T cells and CD86^+^ M1 Mφs) infiltration (**Figure 6A**) and MMP13 and IL-6 expression in the OA synovium (**Figure 6B**). Typical cartilage degeneration (Col I expression, cartilage thinning, and proteoglycan loss) was evident 8 weeks after surgery (**Figures 6C-F**). Overall, M2 Mφs failed to alleviate degenerative progression, with only slight improvement in synovial inflammation. In contrast L-M2a Mφs significantly reduced immune cell infiltration and MMP13/IL-6 expression in the OA synovium (**Figures 6B, C**), reduced Col I expression (**Figure 6D**), and restored tissue integrity, joint space width (**Figure 6F**), and safranin O staining in the cartilage (**Figure 6E**).

We also extracted exosomes from the same number of M2 Mφs (**Supplementary Figure 2C**) to treat OA. However, the overall therapeutic effect was not as obvious as that of L-M2a Mφs (**Figure 6**).

## Discussion

In the past decades, studies on stem cell therapy for OA have been performed globally to evaluate their safety and efficacy and have yielded positive results. They improved the pain, physical function, stability of cartilage defect, and thickening of articular cartilage of OA patients (17). The desired mode of cell therapy in OA involves engraftment of injected stem cells on the chondral defect and repair of cartilage by direct chondrogenic differentiation (18, 19). However, accurate homing and directional differentiation of stem cells are challenging (20). In addition, injected stem cells usually perish rapidly in the joint cavity and are undetectable 14–50 days post-infusion (21, 22). Other studies have shown that the positive effects of therapies involving stem cells are mediated by the secretion of molecules that act in a paracrine manner to restore tissue homeostasis and regulate local immunity (23). It has been proved that the existence of stem cells leads to the production of anti-inflammatory cytokines (such as IL-10 and TGFβ) and TNFα-stimulated gene/protein 6 (TSG-6), which leads to the inhibition of the toll-like receptors-2 (TLR2)/nuclear factor κ-light-chain-enhancer of activated B cells (NFκB) signaling pathway, followed by the downregulation of inflammatory mediators, such as nitric oxide, TNFα, and IL-1β (24, 25). Similarly, upregulation of prostaglandin E2 (PGE-2) and (2,3-dioxygenase) IDO by MSCs leads to the inhibition of IFN-γ, inducing the differentiation of M1-type Mφs to M2-type Mφs (26, 27). However, the immune and inflammatory regulation of stem cells is not as strong as that of immune cells, which makes them insufficient to counteract the long-term chronic inflammatory environment of OA. Moreover, aging and tumorigenicity caused by excessive proliferation and difficulties in obtaining primary stem cells are challenging problems (28, 29).

Recently, Mφs which is a key cell composition of innate immunity and have a powerful role in regulating immunity and maintaining tissue homeostasis have emerged as a novel candidate for cell-based therapy in cancer and regenerative medicine (30). Mφs exhibit numerous potential advantages in the treatment of inflammatory diseases, including OA. First, unlike tissue-resident Mφs, Mφs for therapeutic purposes are based on differentiating a collection of monocytes from blood or extracted bone marrow, which means a stable acquisition method and a sufficient number. Moreover, Mφs, as terminally differentiated and non-proliferative cells, do not have the possibility of reproductive senescence and tumorigenesis during treatment. Furthermore, as a major immunoregulatory cell, Mφs are characterized by an intrinsic and powerful immune and inflammatory regulatory capacity (31). Thus, Mφs are promising seed cells for OA treatment. Based on this, we used Mφ in this study for the first time for OA treatment and obtained encouraging preliminary results, although there are still some limitations and problems that need to be addressed by further studies.

In contrast to M1-based therapy, which is commonly used in antitumor fields, M2-based cell therapy is used in anti-inflammatory and regenerative fields. M2 Mφs show diverse gene expression signatures, and distinct M2a, M2b, M2c, and M2d Mφs subpopulations have been identified using transcriptome analysis (**Figures S3D, E**). It is recognized that M2a Mφs, which are stimulated by IL-4 and characterized by the expression of CD206 and arginase (ARG)-1, present a potent anti-inflammatory function by producing IL-10, IL-1Rα, CCL18, and TGF-β, and pro-regenerative factors such as TGF-β, IGF, and FGF (32, 33). Therefore, in recent years, cell transplantation of M2a Mφ has been used to treat several inflammatory and traumatic conditions with satisfactory results. In a phase 2B clinical trial of 125 patients with dilated cardiomyopathy, compared with the placebo group, intracardiac administration of the mixture of M2 Mφs and mesenchymal stem cells resulted in a decrease in cardiac adverse events (34). Lu et al. also showed that in the mouse model of chronic nephropathy induced by adriamycin, M2a Mφs was injected into tail vein once after 5 days, which prevented kidney injury after 28 days, but inactivated Mφs had no effect. They also revealed that M2a Mφs inhibited effector T cells through the anti-inflammatory effects of cytokines IL-10 and TGF-β, resulting in less tissue damage and inflammation and less fibrosis (35). Although promising results have been observed in other studies, Mφ transplantation did not produce a sufficient therapeutic effect or even aggravated the damage *in vivo*. In an independent study of patients who received the following treatment, intramyocardial delivery of unamplified autologous bone marrow mononuclear Mφs did not show clinical improvement of ischemic cardiomyopathy (36). Moreover, wound administration of mouse Mφs activated IL-4 or IL-10 *in vitro* into the M2-like phenotype damaged skin wound healing in a diabetic mouse model (37). Additionally, adoptive metastasis-inhibitory BM-M2 Mφs could not prevent inflammatory kidney injury (12). It is believed that the poor curative effect in these studies is more likely due to the plasticity of Mφs, leading to a short M2 anti-inflammatory phenotype maintenance *in vivo*. In this study, we observed that unmodified M2 Mφs could not maintain its anti-inflammatory phenotype *in vivo* and even transformed into pro-inflammatory M1 Mφs in an OA environment, which largely compromised its therapeutic effects for OA. Therefore, it is essential to modify Mφs to obtain a stable *in vivo* phenotype.

Based on this, reprogramming of Mφ polarization has become the focus of research. Reprogramming intra-articular Mφs from M1 to M2 by local or systemic delivery of bioactive factors was confirmed to be able to improve the microenvironment of OA to some extent (38, 39). The traditional method of Mφ reprogramming involves changing the polarity of signaling molecules (such as cytokines, receptor agonists, and inhibitory antibodies). For example, Mφ pretreated with LPS + IFN-γ can reduce fibrosis formation after trauma or ischemic injury (40). However, such reprogramming is often transient and insufficient to resist the chronic inflammatory environment *in vivo*. In contrast, genome editing can achieve the long-term effect of complete or partial inhibition of a specific gene by forming stable modifications. Ad5f35, a chimeric adenoviral vector, synergizes with chimeric antigen receptor (CAR) activity, activates the Mφ inflammasome, and provided a beneficial proinflammatory priming signal, which renders CAR-Mφ locked into an M1 phenotype (41). Using this strategy, the selection of target genes is the first step in gene editing. A network typing based on the Mφ phenotype transcriptome found that, unlike the classic M1 Mφ phenotype activated by LPS or IFNγ signaling in acute inflammation, TNF is the key signal that induces Mφs to polarize towards M1 in a chronic inflammatory environment (42). Moreover, TNF is recognized as the most potent factor against the M2 phenotype because of its direct effects on Mφs and the indirect inhibitory effects of TNF on IL-4 and IL-13 production by other innate cell types. Furthermore, IL-4/STAT6 is considered the most important pathway of M2a polarization, and the absence of IL-4, IL-4R, and STAT6 leads to the complete loss of the M2a phenotype (3). In addition, in this study, we also compared TNFR knock-out with IL-4 over-expression and their synergy in pre-experiment, and found that the combination of these two editing was more resistant to OASF (**Figure S3A**). Therefore, it is reasonable to ‘lock’ Mφ in an M2a phenotype in a chronic inflammatory environment by simultaneously knocking-out TNFR1 and up-regulating IL-4.

Appropriate editing method is an important factor in gene editing. Primary immune cells such as monocytes and Mφs have low replication ability and high sensitivity to foreign nucleic acids and viruses. This makes conventional editing methods, such as transfection or lentivirus infection difficult (43). The direct delivery of Cas9 protein and its related single instruction RNA (sgRNA, together comprising the CRISPR-Cas9-RNP) complex provides an attractive alternative method for genome editing based on CRISPR. Protein-mediated strategies are short-lived, thus limiting genome exposure to editing mechanisms, which may lead to unnecessary off-target editing. Transient protein delivery also reduces the risk of immunogenicity in the host due to the continuous expression of active Cas9 (44, 45). Recent studies showed that the efficiency of the Cas9-RNP editing strategy was far superior to that of the traditional method in myeloid cells. They proved that the delivery of Cas9-RNP complex usually produces single or multiple target genes with > 90% KO, so that it is possible to quickly evaluate the functionally deficient genes of donors or cells from a predetermined genetic background without damaging the normal cell function. This process produces genetically edited Mφs, which retains transcripts and protein markers of myeloid differentiation and phagocytic function (14, 46).

**Figure 7:**
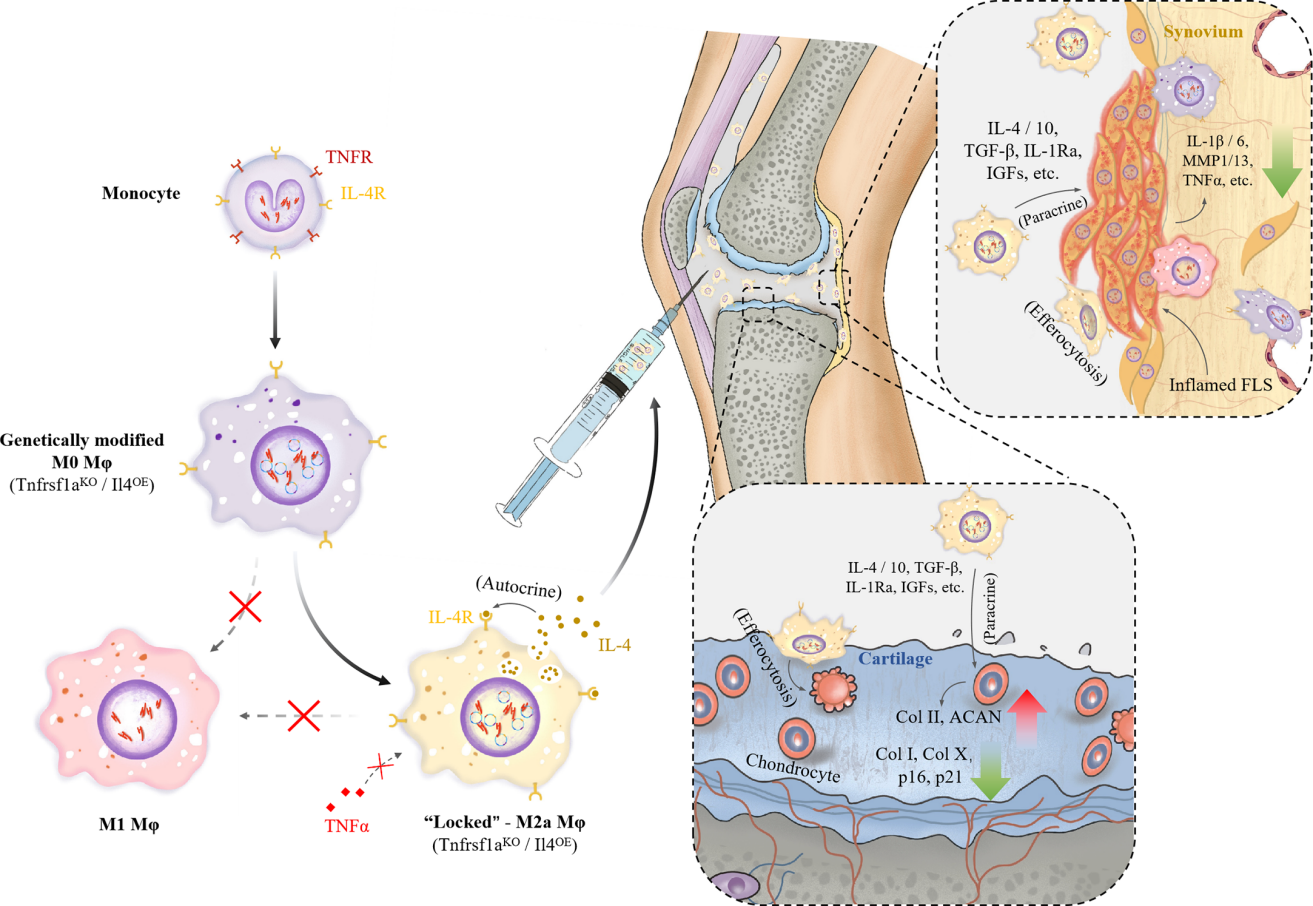
Diagram of construction of L-M2a Mφs and mechanism of its function in joints.

In conclusion, in this study, we constructed locked M2a Mφs with TNFR1 knockout and IL-4 overexpression for OA treatment. We found that it maintained a more stable M2a polarization and anti-inflammatory phenotypes in an inflammatory environment *in vitro* and in a rat OA model. Moreover, an intra-articular injection of L-M2a Mφ delayed the process of OA (**Figure 7**). This study provides insights into the treatment of inflammatory joint diseases including OA.

## Data availability statement

The original contributions presented in the study are included in the article/**Supplementary Material**. Further inquiries can be directed to the corresponding author.

## Ethics statement

The studies involving human participants were reviewed and approved by Institutional Review Board (IRB) of the Third Xiangya Hospital, Central South University (No. 2020-S221). The patients/participants provided their written informed consent to participate in this study.The animal study was reviewed and approved by Department of Laboratory Animals of the Central South University (Changsha, China).

## Author contributions

CL contributed to conception, design, data acquisition, analysis, and interpretation. SW, GX, JH, ZW and WZ contributed to the data interpretation, critically revised the manuscript. XC contributed to conception, design, and data interpretation, drafted the manuscript. All authors read and approved the final manuscript.

## Supplementary material

Supplementary Figure 1 (A) Validation of knockout efficiency of sgTNFR1-EGFP-Cas9-RNP and overexpression of pCDNA -IL-4 in human-derived macrophages by flow cytometry, DNA cleavage assay and Western blot. (B) The isotype controls and gating strategy of flow cytometry in Fig 1E. (C) The PCA plot (left) and Pearson correlation (right) among M2 (A group), M2+OASF (B group), L-M2a (C group), L-M2a+OASF (D group). Mφ: macrophage; L-M2a Mφ: locked M2a macrophage; OASF: OA synovial fluid.

Supplementary Figure 2 (A-B) Polarized phenotype and inflammatory phenotype of M2 and L-M2a Mφ co-cultured with or without OA chondrocytes for 1 or 7 days using western bolt (A, n=3) and RT-qPCR (B, n=5). (C) Nanoparticle Analysis of M2 macrophage exosomes.

Supplementary Figure 3 (A) Comparison of polarized phenotype and inflammatory phenotype among M2 Mφs constructed with Tnfrsf1a knockout and Il4 stimulation (Tnfrsf1a^KO^ group), Il4 overexpression (Il4^OE^) or Tnfrsf1a knockout and Il4 overexpression (Tnfrsf1a^KO^Il4^OE^) when stimulated with or without OASF by RT-qPCR. (B) Comparison of polarized phenotype among M1, M2 and Tnfrsf1a^KO^Il4^OE^ M1 Mφs by Western blot. (C) Expression of CD86 and CD206 in M0, M2, and L-M2a Mφ when stimulated with or without LPS+IFNγ by Western blot. (D) A heatmap of anti-inflammatory and pro-regenerative cytokines expression of M0, M1, M2a, M2b, M2c Mφs by RT-qPCR. (E) Expression of inflammatory factors of chondrocytes co-cultured among with M0, M1, M2a, M2b, M2c Mφs. (F) The expression of CD86 and CD206 in M1, M2 and macrophages treated with OASF. KO: knockout; OE: overexpression; Mφ: macrophage; OASF: OA synovial fluid.

Supplementary Table 1 The source and identifier of reagents used in this study.

Supplementary Table 2 The rat primer sequence for RT-PCR.

Supplementary Table 3 Basic information (gender, age, K/L grade) of 5 OA patients for OASF. K/L grade: Kellgren & Lawrence grade.
