# Supplementary figures and images for: Engineered M2a macrophages for the treatment of osteoarthritis
Source: Front Immunol. 2022 Dec 13;13:1054938. doi: 10.3389/fimmu.2022.1054938 (PMC9792488; doi:10.3389/fimmu.2022.1054938)

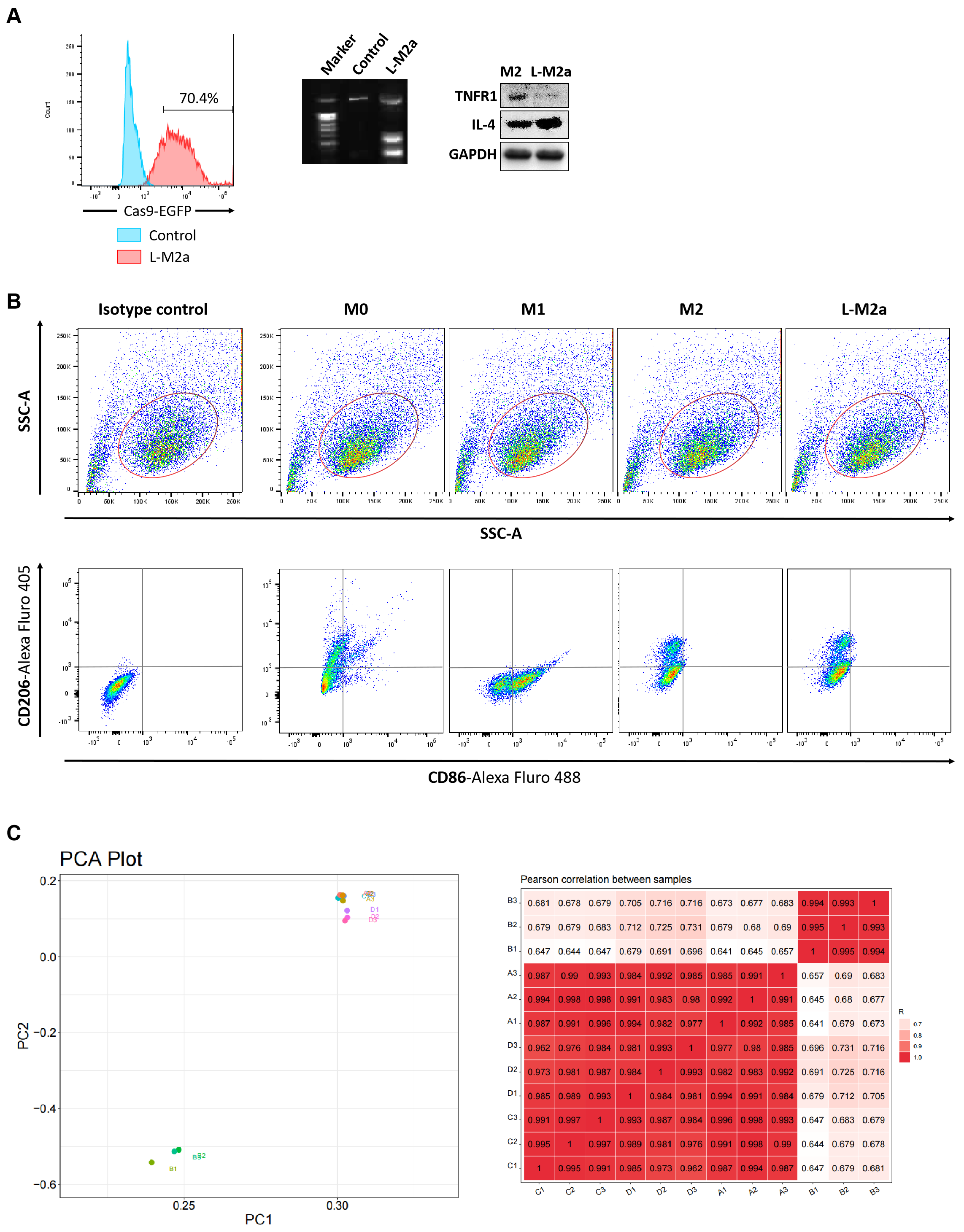

Supplement: Supplementary Figure 1 — (A) Validation of knockout efficiency of sgTNFR1-EGFP-Cas9-RNP and overexpression of pCDNA -IL-4 in human-derived macrophages by flow cytometry, DNA cleavage assay and Western blot. (B) The isotype controls and gating strategy of flow cytometry in Fig 1E. (C) The PCA plot (left) and Pearson correlation (right) among M2 (A group), M2+OASF (B group), L-M2a (C group), L-M2a+OASF (D group). Mφ: macrophage; L-M2a Mφ: locked M2a macrophage; OASF: OA synovial fluid. [file Image_1.tif]

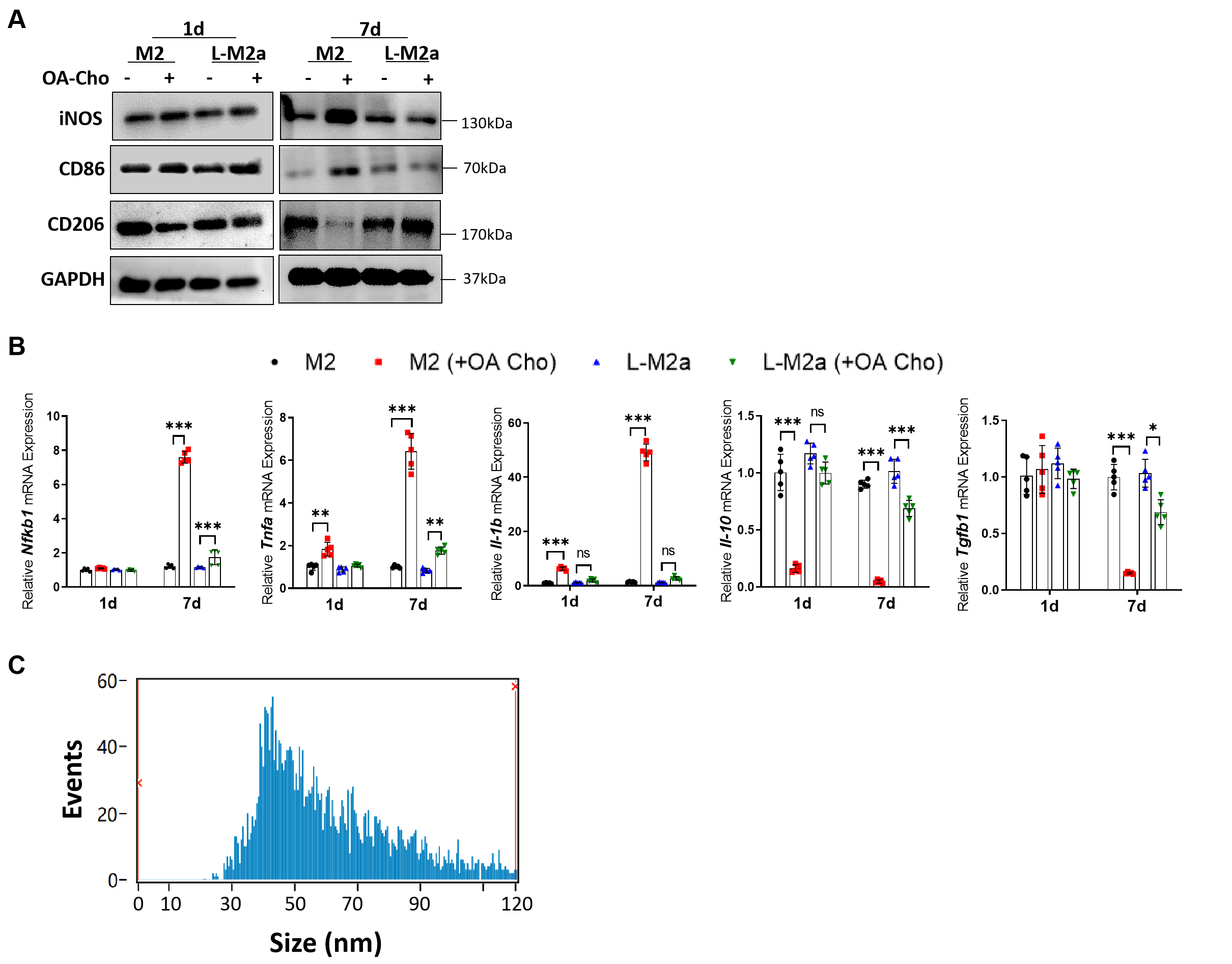

Supplement: Supplementary Figure 2 — (A-B) Polarized phenotype and inflammatory phenotype of M2 and L-M2a Mφ co-cultured with or without OA chondrocytes for 1 or 7 days using western bolt (A, n=3) and RT-qPCR (B, n=5). (C) Nanoparticle Analysis of M2 macrophage exosomes. [file Image_2.tif]

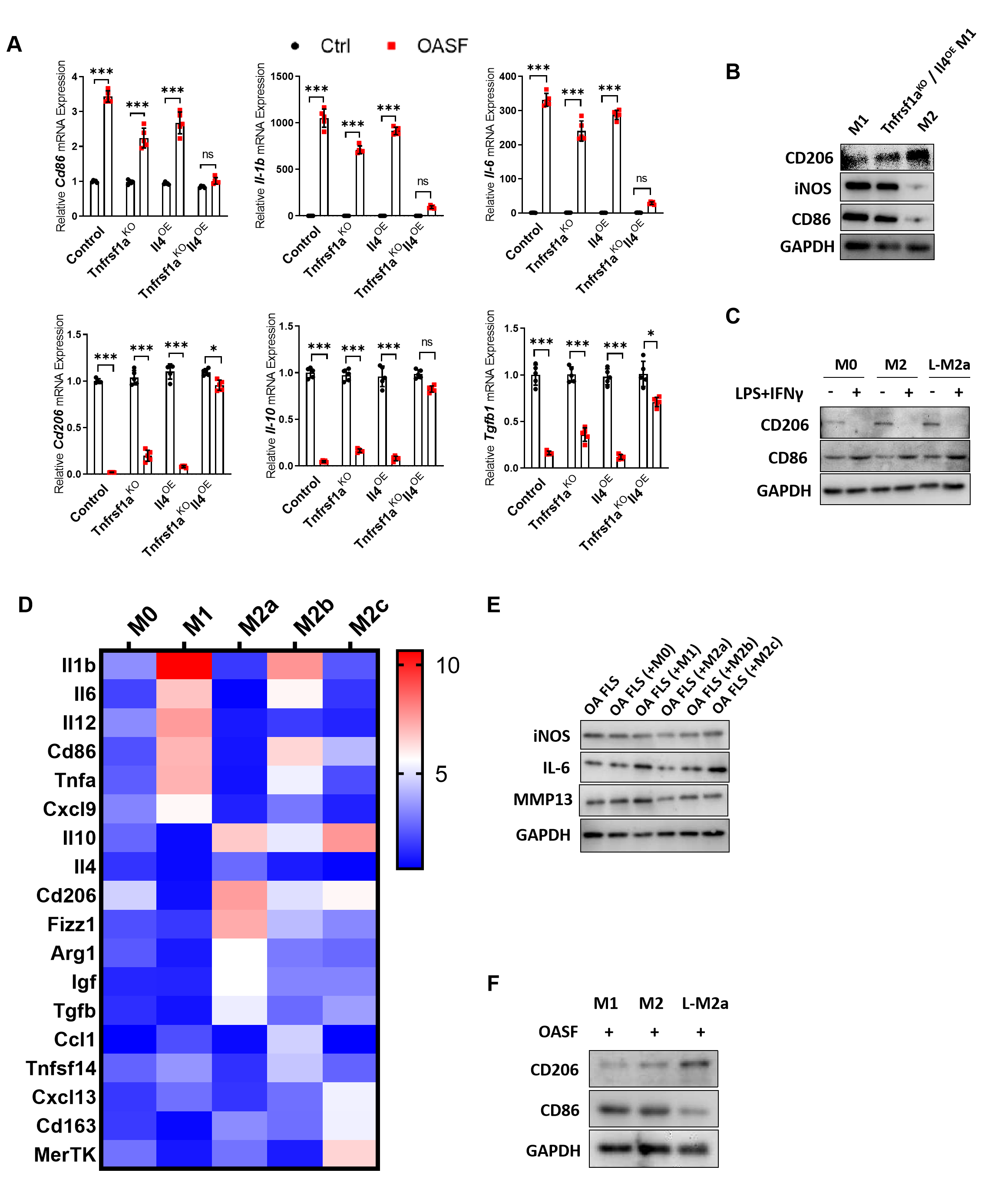

Supplement: Supplementary Figure 3 — (A) Comparison of polarized phenotype and inflammatory phenotype among M2 Mφs constructed with Tnfrsf1a knockout and Il4 stimulation (Tnfrsf1aKO group), Il4 overexpression (Il4OE) or Tnfrsf1a knockout and Il4 overexpression (Tnfrsf1aKOIl4OE) when stimulated with or without OASF by RT-qPCR. (B) Comparison of polarized phenotype among M1, M2 and Tnfrsf1aKOIl4OE M1 Mφs by Western blot. (C) Expression of CD86 and CD206 in M0, M2, and L-M2a Mφ when stimulated with or without LPS+IFNγ by Western blot. (D) A heatmap of anti-inflammatory and pro-regenerative cytokines expression of M0, M1, M2a, M2b, M2c Mφs by RT-qPCR. (E) Expression of inflammatory factors of chondrocytes co-cultured among with M0, M1, M2a, M2b, M2c Mφs. (F) The expression of CD86 and CD206 in M1, M2 and macrophages treated with OASF. KO: knockout; OE: overexpression; Mφ: macrophage; OASF: OA synovial fluid. [file Image_3.tif]
